# The absurdist approach to unveiling possible paradoxical thinking for innovative socio-psychological research

**DOI:** 10.1016/j.mex.2026.103910

**Published:** 2026-04-10

**Authors:** Minh-Hoang Nguyen, Manh-Tung Ho

**Affiliations:** a Centre for Interdisciplinary Social Research, Phenikaa University, Hanoi, Viet Nam; b Institute of Philosophy, Vietnam Academy of Social Sciences, Hanoi, Viet Nam

**Keywords:** Absurdity, Uncertainty, Philosophical paradox, Low-probability events, Granular Interaction Thinking Theory (GITT), Wild Wise Weird

## Abstract

In the age of information abundance, social and psychological sciences face a profound challenge: interpreting and discussing values, meanings, and knowledge that lie beyond data. This paper proposes an epistemic framework termed the absurdist approach to unveiling possible paradoxical thinking, grounded in the principles of Granular Interaction Thinking Theory (GITT) and enriched by self-reflection through imaginative literary and philosophical paradoxes that are exemplified by *Wild Wise Weird*—a collection of fables that intertwine innovative storytelling with contemporary sensibilities, offering both moral insight and moments of self-reflection. Specifically, *Wild Wise Weird* illustrates, in minimalist doses of self-reflective humor, how events of negligible probability can nonetheless possess deep significance, thereby complementing GITT’s explanatory mechanisms by completing the cognitive cycle of interpretation and reflection. Through this synthesis, the absurdist approach encourages intellectual humility in constructing arguments and interpretations, prompting scholars to look beyond data and statistical findings toward the epistemic depths of human reasoning. Ultimately, this combination represents an epistemological application that strengthens methodological practice. It enhances the analytical power of both quantitative and qualitative research while mitigating the risks of overgeneralization—yet still recognizing and valuing low-probability patterns that often carry profound insight across disciplines, including psychology, sociology, healthcare, environmental studies, politics, and economics.

• The paper proposes a thinking method that integrates the theoretical reasoning power of GITT with counter-assumptive propositions found in literary and philosophical paradoxes, exemplified by *Wild Wise Weird*.

• This combination enables deeper exploration of humanistic values and the recognition of low-probability patterns often overlooked in socio-psychological sciences.

• As an epistemological approach, it enhances both quantitative and qualitative research by expanding interpretive capacity while reducing the risks of overgeneralization.

## Graphical abstract

**Figure:**
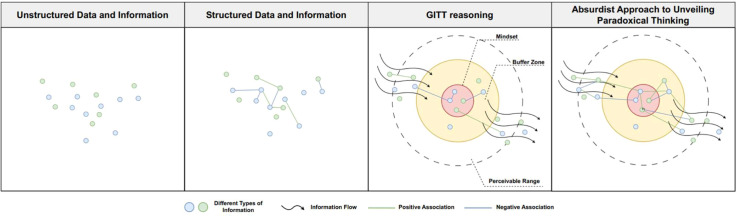

## Specifications table

**Table:** 

**Subject area**	Psychology
**More specific subject area**	Social sciences Psychological sciences
**Name of your method**	Absurdist Approach to Unveiling Possible Paradoxical Thinking
**Name and reference of original method**	Vuong, Q.-H., & Nguyen, M.-H. (2024b). Exploring the role of rejection in scholarly knowledge production: Insights from granular interaction thinking and information theory. *Learned Publishing*, 37(4), e1636. https://doi.org/10.1002/leap.1636 Vuong, Q.-H. (2024). *Wild Wise Weird*. AISDL. https://books.google.com/books?id=N10jEQAAQBAJ Vuong, Q.-H., Nguyen, M.-H., Ho, M.-T., & La, V.-P. (2025). *GITT: Essentials, Uses, and Usage*. AISDL. https://books.google.com/books?id=FK-EEQAAQBAJ
**Resource availability**	Not applicable

## Background

With the emergence of digital spaces, social media, advanced data collection, and artificial intelligence, the world has entered an era of information and data abundance. This proliferation of data increases uncertainty within systems of thought and value. Consequently, there is a growing need to look through data to perceive the informational interactions that shape values, meaning, and knowledge in scientific inquiry. This paper proposes a method termed the *absurdist approach* to unveiling possible paradoxical thinking, grounded in the principles of Granular Interaction Thinking Theory (GITT) and self-reflection, which are deeply embedded in both science and philosophical works, such as *Nan Hua Jing* [1], *Wild Wise Weird* [2], and *Kingfisherish Wandering* [3].

In this method, GITT is used as the theoretical foundation for constructing models, providing reasoning for the results, and serving as a framework to connect the results with preexisting knowledge, facts, data, or information. While GITT provides the general mechanisms and logical foundation for explaining how values emerge from informational interactions, it remains limited in addressing the qualitative dimension—namely, the emergence of humanistic or low-probability values within this process. Using*imaginative* literary and philosophical paradoxes through artistic and reflective experiences, exemplified by *Wild Wise Weird*—a collection of fables that weave innovative storytelling with contemporary sensibilities, offering both life lessons and moments of self-reflection—can help fill these gaps and address this issue [2].

In the next section, we present GITT’s theoretical details and its prior applications, as well as how the literary and philosophical paradoxes in *Wild Wise Weird* can help complement GITT’s reasoning power. Finally, we provide several remarks on the method as well as its limitations.

## Method details

As the method includes two primary components: 1) GITT theoretical reasoning and 2) literary and philosophical paradox integration.

### GITT theoretical reasoning

For implementing this method, understanding GITT is a prerequisite. GITT, rooted in the principles of quantum mechanics, Shannon’s information theory, and mindsponge theory, posits that macroscopic reality emerges from the interactions of quanta at the microscopic level [[4], [5], [6], [7], [8]]. Its framework and principles are also mathematically grounded in probability theory and set theory, enabling the formalization of complex socio-psychological dynamics through mathematical modeling. Such formalization enhances conceptual precision and logical rigor while fostering interdisciplinary convergence with established knowledge in the natural sciences [9,10,8]. More broadly, GITT aligns with a long intellectual tradition that adapts conceptual tools from the physical sciences to illuminate socio-economic reasoning and human cognition. Adam Smith, often regarded as the father of modern economics, was influenced by Newtonian mechanics and the mathematical rigor of early calculus [11,12]. Subsequently, Leon Walras formalized general equilibrium theory [13]; Adolphe Quetelet advanced the idea of “social physics” [14]; Louis Bachelier proposed that stock prices follow a random walk—anticipating the mathematical structure of Brownian motion later formalized by Albert Einstein [15]; and this trajectory also result in the derivation of the Black–Scholes equation in the early 1970s, which provided closed-form solutions for option pricing [16].

GITT conceptualizes existence through two interdependent and co-constitutive dimensions: the mind and the environment. The mind functions as an information-gathering and -processing system, while the environment—encompassing societies, ecosystems, the climate, and the Earth as a whole—operates as a broader, multi-layered informational network. A central notion derived from quantum mechanics, relationality, asserts that all phenomena arise from interactions and that the variable properties of any object exist only in relation to others. Within this relational framework, the mind and its surroundings are engaged in a continual exchange of information, dynamically reorganizing to sustain coherence and adaptability. Systems that regulate these exchanges effectively are those capable of maintaining and advancing their existence. In other words, survival and evolution within a dynamic world depend on the efficient management of information—its acquisition, storage, transmission, and processing [17,18]. This view aligns with Darwinian evolutionary theory, which identifies adaptation as the essential condition for persistence [19,20].

As minds interact with increasingly complex environments, the amount, heterogeneity, and speed of incoming information intensify. This proliferation, when exceeding the mind's existing predicting models, raises informational entropy, understood here as uncertainty or the expected level of “surprise” associated with potential outcomes. Informational entropy is formally defined by Shannon’s entropy function [6]:

$$H\left(X\right)=-\sum_{i=1}^{n}P\left(x_{i}\right)\log_{2}P\left(x_{i}\right)$$

where H(X) represents the entropy of a random mind X with the set of possible cognitive interpretations, beliefs, or values $\left\{x_{1},x_{2},\ldots ,x_{n}\right\}$ and corresponding probabilities that the mind assigns to that option $\left\{P\left(x_{1}\right),P\left(x_{2}\right),\ldots ,P\left(x_{n}\right)\right\}$. Entropy increases as the mind, when lacking sufficient information to differentially weight possible outcomes, assigns equal probability to all possible outcomes, i.e., $P\left(x_{i}\right)=\frac{1}{n}$. At this point, uncertainty is highest: no single outcome dominates, and in adaptive terms, this state can manifest as decision paralysis, cognitive overload, or value instability.

Just as quanta, atoms, and molecules constitute the structural and functional basis of cells—the fundamental units of life [21,22]—the human mind’s cognitive operations can likewise reflect the foundational characteristics that define the quantum world: granularity, relationality, and indeterminacy. These principles help illuminate how humans optimize limited cognitive and energetic resources by evaluating, comparing, assigning probabilistic weight, or even filtering out informational units according to their relevance for survival, growth, and reproduction to reduce entropy. From this perspective, the mind progressively detects which internal informational structures fail to correspond with external reality through ongoing interaction with its environment. Through repeated cycles of engagement, feedback, and updating, misaligned mental representations are filtered out, while those that withstand experiential justification are retained. Over time, the information that endures this iterative process of validation approximates what epistemologists consider a “justified true belief” [23,24]. Meanwhile, values can be interpreted as emergent properties arising from the interaction among diverse informational units within the mind—such as experiences, perceptions, beliefs, emotions, biological predispositions, and worldviews—alongside new inputs from environmental, socio-cultural, and economic contexts. Although knowledge and values are themselves informational entities, they differ from ordinary information in that they possess a higher probability of retention, or, in energetic terms, warrant greater resource allocation for maintenance.

Based on these characteristics, GITT has served as a theoretical foundation and conceptual framework for a growing body of research across multiple disciplines, ranging from African knowledge systems in environmental education [25], water consumption behaviors [26], potable water reuse willingness [27], supply-sourcing strategies for school meal [28], consumers’ willingness to pay for sustainable fashion [29], somatic symptoms and depressive networks [30], social avoidance of adolescents with depressive disorders [31], trust in space tourism [32], etc.

However, even though the mind can lower internal entropy by reorganizing its informational architecture and filtering out waning inputs, this entropy-reduction process remains structurally imperfect. First, uncertainty is not merely a limitation of human measurement but seems to be an ontological feature of reality itself. The Heisenberg Uncertainty Principle shows that certain pairs of physical properties cannot be simultaneously determined with arbitrary precision [33,34]. The more precisely one property is specified, the more indeterminate the other becomes. This indeterminacy is not a temporary epistemic shortcoming but a constitutive characteristic of matter. Second, although human cognition is biologically and evolutionarily oriented toward detecting causality, coherence, and purpose [[35], [36], [37]], it operates under persistent epistemic constraints. These include bounded sensory access, limited memory capacity, and biases shaped by preexisting knowledge structures and value systems. This intrinsic imperfection has a formal analogue in Kurt Gödel’s Incompleteness Theorems [38]. Gödel demonstrated that within any sufficiently expressive formal system, there exist true propositions that cannot be proven using the system’s own axioms. His results imply that even mathematics—arguably the most rigorously structured epistemic system available to us—is fundamentally incomplete.

Consequently, the mind needs to continually confront deviations between its internal informational structures and external reality, making interaction with reality itself a persistent source of uncertainty [39]. Such deviations can be broadly categorized into two forms: stupidity and delusion. Stupidity denotes a condition in which one’s understanding of reality is insufficient or underdeveloped, whereas delusion refers to the maintenance of fundamentally distorted or false representations of reality [40]. As the mind engages with a dynamic and evolving environment, internally coherent (i.e., low-entropy) states that are grounded in distorted beliefs, rigid convictions, or entrenched prejudices become structurally fragile. When confronted with disconfirming evidence, these seemingly stable configurations may collapse abruptly, producing rapid surges in entropy (i.e., uncertainty or the expected level of “surprise”).

From this perspective, uncertainty may evolve into absurdity when newly encountered information cannot be coherently connected to and integrated into existing systems of reasoning. Absurdity can be generally understood as the tension—or collision—between the human drive for systemic coherence (such as reason, purpose, and permanence) and a reality marked by indeterminacy [[41], [42], [43]]. As Camus [42] suggests, the absurd emerges from the “divorce” between the human demand for intelligibility and a world that offers no inherent guarantees of meaning or moral order. Interpreted through an information-processing lens, absurdity arises when incoming information from reality is incompatible with the mind’s prevailing categories of meaning and justification (e.g., beliefs and values)—categories that function precisely to maintain internal coherence and reduce entropy [44].

**Fig. 1:**
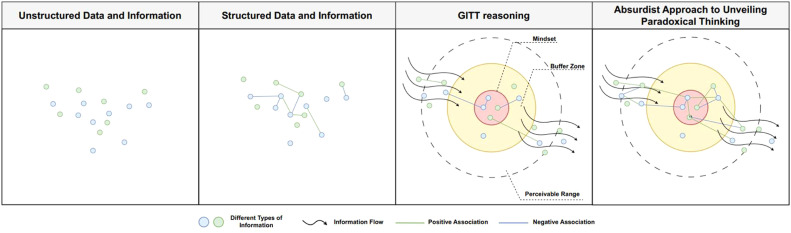
Comparison between the absurdist approach to unveiling possible paradoxical thinking and other interpretation scenarios.

Based on the reasoning above, it is plausible to say that no scientific, mathematical, moral, or metaphysical system—including GITT—can achieve complete self-explanation without appealing to assumptions or principles that extend beyond its own internal logic and explanatory boundaries. Although GITT offers a coherent set of mechanisms for explaining how meaning, beliefs, and values dynamically emerge from informational interactions, its coherence may inadvertently foster a sense of epistemic closure. In practice, this can lead to the systematic down-weighting or dismissal of information that appears incompatible with its core premises. The more rigidly researchers rely on any formal framework, the greater the risk that preexisting epistemic structures will shape what is perceived as plausible, relevant, or even visible. Such reliance may obscure subtle environmental signals or anomalous patterns that resist immediate incorporation into the model or framework. For this reason, GITT—like any theoretical system—benefits from deliberate engagement with counter-assumptive propositions. Reflective confrontation with low-probability, paradoxical, or humanistically rich possibilities helps expose the latent absurdities within an epistemic system (see Figure 1). In doing so, it mitigates premature closure and reopens analytical space for insights that move beyond the preexisting epistemic structures, dominant data patterns, and statistical regularities.

### Literary and philosophical paradox integration

The literary and philosophical paradoxes, exemplified by *Wild Wise Weird*, can help address this limitation. The work provides an allegorical lens through which the circulation of information across minds, societies, and environments can be examined, particularly in relation to the acceptance or rejection of social norms and the confrontation with emergent, seemingly contradictory forms of knowledge. Through its narrative structure, *Wild Wise Weird* demonstrates how events assigned negligible probability may nonetheless carry profound epistemic significance. In doing so, it exposes latent absurdities embedded within established systems of thought and reveals the fragility of seemingly stable epistemic orders. This perspective complements GITT’s general explanatory framework by completing the reflective cycle—extending analysis beyond structural mechanisms toward deeper interpretive engagement [45,46]. The quantitative philosophical analysis of the book’s 48 stories explicitly highlight its demonstration of the dynamic interplay between uncertainty and absurdity, reinforcing its relevance as a counterbalancing lens to formally structured theoretical models [44]. Moreover, *Wild Wise Weird* adopts a distinctive orientation compared to literary and philosophical works that primarily critique political regimes, dominant sociocultural norms, or ideological systems—such as *Animal Farm* [47], *Alice’s Adventures in Wonderland* [48], or *Laugh or Lament* [49]. Although it incorporates certain elements of Vietnamese cultural context, its central concern lies in examining individual information-processing dynamics. This inward focus renders the work relatively ideologically independent and socially and culturally transcendent [45]. In other words, the paradoxes presented in *Wild Wise Weird* are therefore intended not as socially and culturally coded metaphors, but as demonstrations of cognitive mechanisms. Their function is to help readers detect early signals of huge absurdity in their thinking—such as rigid belief reinforcement, speculation, self-sealing reasoning, and the normalization of internal contradictions and logical fallacy. Such characteristics make it particularly well suited for integration as a methodological component.

Furthermore, in a period of escalating uncertainty—shaped by deepening inequality, repeated market failures, AI-driven socio-economic transformations, intensifying political polarization and geopolitical rivalry, and existential risks associated with climate tipping points and the transgression of planetary boundaries—the importance of engaging with philosophical and literary works becomes especially pronounced. Such works illuminate the persistent absurdities embedded in how humans relate to one another and to the Earth. As Dai and Duflo [50] contend, social commentary and literature—such as *Wild Wise Weird*, particularly through its embedded Nature Quotient—can function as “an effective way to bridge the gap between awareness and sustained behavioral change,” because they “encourage emotional and cognitive involvement with ecological issues.”

Specifically, *Wild Wise Weird* can thus be seen as conceptually and philosophically complementing GITT’s model of information processing through several mechanisms:

*First*, the fables frequently depict individuals—or birds as metaphors—who interpret, misinterpret, selectively attend to, or disregard information. These narrative patterns mirror how humans process information in real-world contexts: through cognitive biases, heuristic shortcuts, emotionally laden priors, and culturally embedded interpretive frameworks. By making these processes visible, the stories cultivate metacognitive awareness—a reflective reassessment of one’s own cognitive model concerning the issue at hand—among both researchers and readers. Such reflection can loosen rigid interpretive schemas, create space for low-probability or marginalized possibilities, and promote more adaptive engagement with uncertainty.
*Second,* each story can be understood as a micro-model of communication. It encodes meaning through symbolic structure and metaphor, transmits that meaning through a narrative medium that reader can easily interpret and integrate into their mind. This narrative form expands the capacity for information reception and imagination among both researchers and readers. Not all individuals readily engage with dense, abstract scientific arguments; highly formalized reasoning can create cognitive barriers that limit broader intellectual interpretation. By contrast, narrative structures lower these barriers. They translate abstract concepts into experiential and more relevant scenarios, enabling readers to internalize, simulate, and creatively extend ideas that might otherwise remain inaccessible. Thus, the narrative can function as a complementary epistemic channel—one that enhances interpretive flexibility, stimulates imagination, and fosters the generative development of new insights beyond strictly formal analytical discourse.
*Third*, through imaginative, exaggerated, or seemingly implausible situations, the stories demonstrate how information initially considered to be correct, virtuous, or useful can, under altered conditions, become contradictory or fundamentally misaligned with reality. In doing so, they expose the latent fragility of established epistemic systems—systems that appear coherent and internally consistent yet may conceal unresolved uncertainty. Through this narrative mechanism, readers are invited to simulate scenarios in which a once “sound” framework becomes untenable when confronted with novel forms of information. What initially functions as order can dissolve into absurdity as contextual variables shift, assumptions are inverted, or neglected perspectives surface. These imaginative models help expand readers’ cognitive flexibility. By rehearsing the possibility of epistemic reversal, the stories cultivate preparedness for paradigm disruption and encourage a more humble, adaptive engagement with emerging knowledge [5].
*Fourth*, recognizing absurdity within oneself is crucial for overcoming order-induced blindness—a condition in which historically constructed systems of order (i.e., political, cultural, ideological, or epistemic one) are implicitly treated as self-validating, complete, and immune to updating. When such systems harden into unquestioned structures, they reduce cognitive noise at the cost of perceptual openness, fostering an illusion of coherence while obscuring subtle misalignments with reality. In *Wild Wise Weird*, absurdist moments operate as epistemic resolutions to this blindness. By pushing assumptions to their logical extremes or placing them in exaggerated narrative contexts, the stories expose contradictions embedded within seemingly stable belief systems. This process resembles a *reductio ad absurdum*, in which unjustified beliefs, rigid prejudices, and inherited assumptions are progressively filtered out, while interpretations that better withstand experiential and logical scrutiny are retained. Over time, what endures this iterative cycle of contradiction and revision approximates a form of justified true belief—not as a final state of certainty, but as a continually refined alignment between internal coherence and external reality [18].

Through these four mechanisms, the artistic, philosophical, and reflective experiences in *Wild Wise Weird* can enhance GITT’s explanatory and reasoning capacity—strengthening its ability to interpret constructed models, elucidate estimated results (including seemingly contradictory findings), and extend the analytical scope toward low-probability scenarios that arise from complex and dynamic information-processing yet are often overlooked.

Furthermore, integrating the literary and philosophical paradoxes found in *Wild Wise Weird* into scientific reasoning can reinforce intellectual humility in the formulation of arguments and interpretations. Such integration nurtures balance and composure in reading, reviewing, and articulating the significance of scientific findings—encouraging scholars to reflect beyond data and statistical analyses. In this light, GITT combined with *Wild Wise Weird* becomes an effective approach for cultivating the soundness, moderation, and plausibility of research outcomes.

## Method validation

The method has been operationalized and validated through successful applications in a series of our innovative studies on human–nature relations, such as Nature Quotient [51], the battery bubble [52], non-linear thinking and serendipity [53], peace with nature [54], climate change weaponization [55], the vulnerabilities of artificial environmental protection systems [56], innovation curses [57], etc. It has also informed our inquiries into human–AI nexuses, encompassing topics such as the premises for understanding human–computer interactions [58], the hidden human costs of computer collaborations [59], the intersection between AI and retracted science [60], etc.

The Absurdist Approach generally involves five main steps:

1. Issue identification
2. Formulation of a model/framework using GITT
3. Uncertainty/absurdity/idea identification through literary and philosophical works
4. Integration of uncertainty/absurdity insights into the model/framework
5. Validation of the updated model/framework

It should be noted that the five steps may be applied partially or fully, and their sequences can be changed depending on the research objective and the intended role of literary or philosophical engagement. In the first example, we show how a story is operationalized through these five steps, whereas the second example will demonstrate how these steps are partly used to inspire statistical modeling.

### Example 1: Innovation curse

The study titled “Innovation curse: The wastefulness of technologies believed to mitigate climate change” serves as a demonstrative case of how the Absurdist Approach to Unveiling Possible Paradoxical Thinking is operationalized in practice [57]. Specifically, the method involves five main steps:

#### Step 1: Issue identification

The first step involves identifying the focal issue. In this case, we examined how humanity addresses climate change and environmental degradation. Through extensive review, we observed that technological climate solutions have become the dominant strategy—often overwhelmingly prioritized over nature-based solutions. Accordingly, the study explicitly focused on technological climate solutions, aiming “to examine how the proliferation of technological climate solutions contributes to the innovation curse and accelerates the erosion of nature-based solutions and Indigenous and Local Knowledges.”

#### Step 2: GITT-based framework construction

In the second step, we formulated a GITT-based framework to examine how information processing—particularly knowledge selection in complex decision-making environments—systematically prioritizes technological climate solutions.

Grounded in GITT, we conceptualized humanity as a collective information-processing system that needs to filter environmental ideas, knowledge, and data to survive and adapt. Within the context of climate change, the proliferation of proposed solutions creates a high-entropy informational environment, activating cognitive, social, and institutional filtering mechanisms. Decision-makers confront a vast solution landscape, ranging from high-tech interventions such as carbon capture and storage, geoengineering, and AI-driven monitoring systems, to nature-based alternatives rooted in Indigenous and Local Knowledges (e.g., traditional agroforestry and community-led conservation). Under such conditions, filtering decisions rely heavily on pre-existing core values embedded within institutional frameworks, economic models, and cultural assumptions.

These core values exhibit two key attributes:

• Preference for proprietary, patentable technologies aligned with market incentives and scalable profitability.
• Privileging of formalized Western scientific approaches compatible with bureaucratic, academic, and policy structures.

As a result, information aligned with technological progress and perpetual economic growth is readily absorbed, while knowledge that challenges these values—particularly non-proprietary, place-based Indigenous and Local Knowledges—is marginalized. The system thus maintains internal coherence while addressing environmental crises in ways that simultaneously reinforce technological expansion and economic development.

Within a conventional epistemic framework, such an explanation may be deemed valid and serve as a benchmark for subsequent research and policy decisions. However, under the Absurdist Approach, explanatory adequacy must also be tested through interaction with insights from literary and philosophical works to examine whether the framework generates latent absurdities—particularly under alternative epistemic lenses or low-probability conditions.

#### Step 3: Absurdity identification through literary works

*Wild Wise Weird* provides narrative material that renders potential absurdities accessible and intuitively graspable. Notably, its story titled “Innovation” directly parallels the issue under investigation.

In the story, Kingfisher, the protagonist of the book, develops an innovation intended to conserve energy while maintaining territorial protection in the technological age. He successfully invents a device enabling him to inflate his belly defensively and dart at high speed at the press of a button. The village greatly admires his technological achievement.

However, no genuine threat arises. Out of boredom, Kingfisher repeatedly activates the device during routine fishing, transforming innovation into ritualized technological performance. When a gentle visitor—Young Belted Kingfisher—arrives, Kingfisher interprets him as an enemy and escalates the technological display. Despite increasingly frantic demonstrations, other birds in Bird Village only see him as “a circus clown,” while the the visitor remains unfazed:

“From that moment on, the entire Bird Village sees Kingfisher inflating his belly and darting quickly, then slamming on the brakes and deflating his belly. Then inflating the belly, flying in circles, and deflating again... Pressing those buttons faster and faster, Kingfisher resembles a circus clown.

But there is one thing he can’t achieve: fear.”

Ironically, the “invading enemy” ultimately thanks Kingfisher for what he perceived as a welcoming dance:

“– Dear Respectable Kingfisher. Thank you for the fantastic folk dance performance to welcome me to the Bird Village. Now, can you please help me find the way to the neighboring Heron Village near Crab Mountain?”

The story reveals a central absurdity: technological innovation may achieve functionality, admiration, and institutional validation while remaining misaligned with the actual problem it purports to solve. In this case, innovation becomes self-referential performance rather than a meaningful solution.

#### Step 4: Integrating absurdist insights into the framework

These absurdist insights allowed us to refine the GITT-based framework. In the updated model, the dominance of technological climate innovations is conceptualized as a low-entropy lock-in effect. The system’s strong prior commitments to technological and economic paradigms reduce informational diversity, narrowing the perceived solution space.

**Fig. 2:**
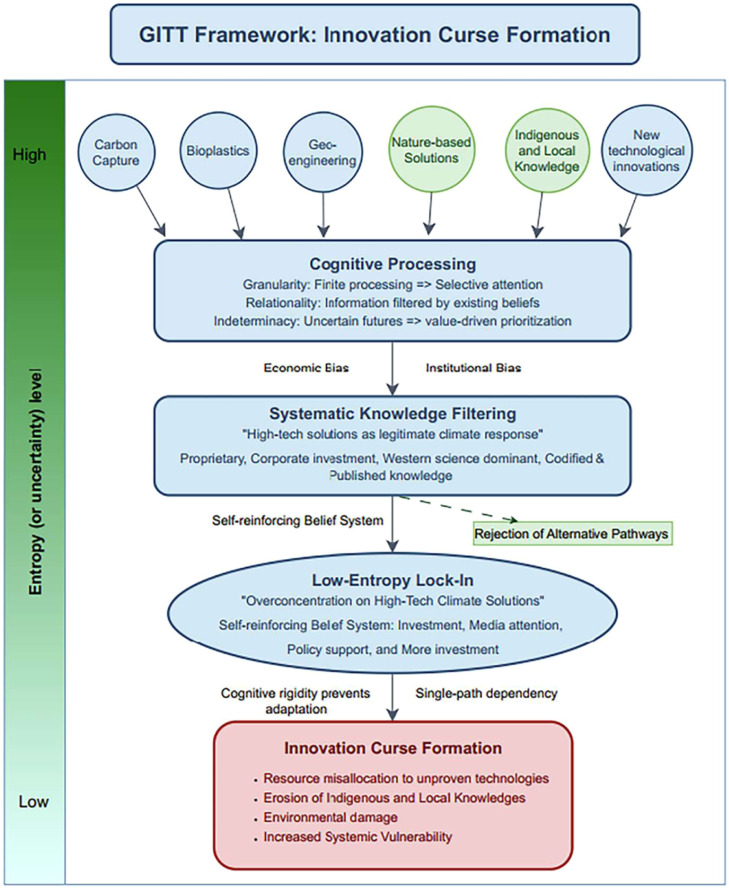
GITT-based framework of the innovation curse formation. Retrieved from Nguyen, Gagnon, et al. [57] under CC BY 4.0.

Rather than enabling adaptive flexibility, technological climate solutions may reinforce systemic path dependency, concentrating attention and resources on high-tech interventions despite empirical limitations. This lock-in effect constrains adaptive updating in response to emerging evidence supporting nature-based solutions. Fig. 2 illustrates how a counterproductive lock-in effect narrows the solution space and contributes to the formation of an innovation curse—defined as a paradoxical condition in which costly, energy-intensive technological interventions are persistently prioritized despite uncertain or limited effectiveness, thereby exacerbating environmental challenges while crowding out more viable and context-appropriate alternatives.

#### Step 5: Updated framework validation

In the final step, we validated the absurdist GITT-based framework by applying it to real-world technological cases, including Carbon Capture and Storage, bioplastics, and glacier geoengineering. Across cases, these technologies demonstrate high costs, inefficiencies, and long-term risks, yet continue to receive disproportionate economic and institutional support relative to lower-cost, time-tested nature-based solutions. These results, along with other empircal evidence shown in the study, validate the updated framework.

We included a quotation from the “Innovation” story at the beginning of the paper to highlight the intellectual inspiration underpinning the study and to serve as a cognitive catalyst, inviting readers to pause, contemplate, and reflect beyond the strictly scientific content presented in the paper. For full context of how the Absurdist Approach is operationalized, readers can refer to the study published in *Humanities and Social Sciences Communications* [57].

### Example 2: In search of value

The study titled “In search of value: The intricate impacts of benefit perception, knowledge, and emotion about climate change on marine protection support” provides a representative demonstration of how *Wild Wise Weird* can inspire complex, non-linear statistical modeling [29]. The modeling process unfolded in three steps.

#### Step 1: Idea identification through literary works

The story “Light and Free” in *Wild Wise Weird* served as the intellectual catalyst for the statistical modeling in Nguyen, Duong, Nguyen, et al. [29]. In the story, Kingfisher and Cuckoo visit a paddy field near harvest season and encounter a strange thing. On one side of the field, the plants are tense and unresponsive:

“Some plants are busy flexing their muscles to counter the wind, while others are struggling to keep their heads intact or completely occupied with fighting off the rapacious birds. With their backs bent and faces down, no one is in the mood for idle chitchat.”

When the two birds move to the other side, however, the plants appear cheerful and talkative. Curious, Kingfisher asks why two groups of plants in the same field behave so differently. A Paddy Youth responds:

“– Sir, it’s because our circumstances are different. Our bunch is light and free, while those guys spend all day long worried and guarding their ripe grains. What a terrible waste of time! Beautiful sunny days are for singing, dancing, and chattering away…”

Yet Kingfisher quickly notices that these seemingly carefree plants are, in fact, crooked. The crucial insight is that although the plants are from the same species and share the same environment, their attitudes differ depending on whether they have ripe grains to protect. In other words, their objects of care condition their orientation toward the world.

This insight inspired us to examine whether human attitudes and behaviors toward environmental protection might likewise depend on what individuals perceive as threatened or valuable. Importantly, the story suggested that such relationships may rather be non-liear, conditional, and context-dependent than linear.

#### Step 2: Integrating insights into model construction

Building on this narrative insight, we considered how it could inform our research on the human–nature nexus—specifically, how individuals think about, respond to, and support actions addressing climate change and biodiversity loss.

We therefore constructed a three-way interaction model to examine the interplay among 1) perceived benefits of aquatic ecosystems in climate change reduction, 2) knowledge about climate change, and 3) emotion toward climate change, in predicting support for marine protection. The statistical formulation is as follows:

(1.1) SupportforOcean∼normal(μ,σ)

(1.2) $$\begin{array}{c} \mu_{i}=\beta_{0}+\beta_{1\;}*Benefits\_ClimatechangeReduction_{i}+\beta_{2}*KnowledgeTowardClimateChange_{i}+\beta_{3}*\\ KnowledgeTowardClimateChange_{i}*Benefits\_ClimatechangeReduction_{i}+\beta_{4}*\\ EmotionTowardClimateChange_{i}+\beta_{5}*EmotionTowardClimateChange_{i}*\\ Benefits\_ClimatechangeReduction_{i}+\beta_{6}*EmotionTowardClimateChange_{i}*\\ Benefits\_ClimatechangeReduction_{i}*KnowledgeTowardClimateChange_{i} \end{array}$$

(1.3) β∼normal(M,S)

**Fig. 3:**
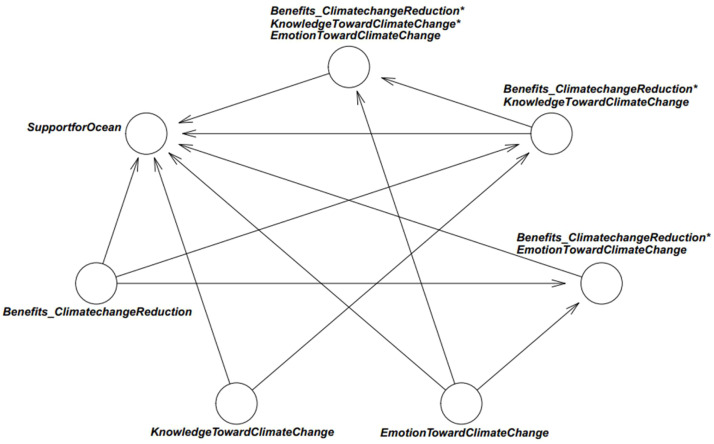
Model’s logical network. Retrieved from Nguyen, Duong, Nguyen, et al. [29] under the authors’ copyright.

This specification represents a moderated multiple regression with both two-way and three-way interactions, enabling us to test whether the effects of benefits, knowledge, and emotion are mutually conditional. The model’s logical network is shown in Fig. 3. A detailed theoretical justification grounded in mindsponge theory (i.e., GITT) is presented in the study’s Theoretical Foundation section.

#### Step 3: Model validation and result interpretation

To test the model, we employed Bayesian Mindsponge Framework (BMF) analytics, integrating GITT-based reasoning with Bayesian inference [61]. The analysis was conducted on data from 709 stakeholders across 42 countries, collected within the European Commission H2020 project Marine Coastal Ecosystems Biodiversity and Services in a Changing World (MaCoBioS) [62].

At first glance, the observed interaction patterns may appear paradoxical, or even absurd, under linear theoretical frameworks that assume additive and monotonic relationships among perception, knowledge, emotion, and behavior. However, when interpreted through GITT and the insight from “Light and Free,” the results become theoretically coherent and closely aligned with the concept of “objects of care”—valued entities such as people, places, identities, or goals that individuals seek to preserve [63]. Through this lens, we interpreted the empirical findings as reflecting conditional cognitive processing: the effects of perception, knowledge, and emotion are not independent or additive, but mutually contingent. In line with GITT’s multi-filtering mechanism, information is absorbed, evaluated, and prioritized based on its perceived relevance to one’s objects of care. Consequently, support for marine protection emerges from dynamic interactions among these informational components rather than from linear relationships.

An explanatory passage based on mindsponge theory (i.e., GITT) presented in the study is provided below:

“According to the Mindsponge Theory, the observed findings can be clarified by the variation in information vitality during the information processing. In this context, an individual's emotional responses to climate change do not directly stem from the climate change itself but rather from the perception that their “objects of care” are endangered by it [63]. Consequently, an increased sense of concern or worry about climate change may indicate a heightened significance of these “objects of care” to the person. These “objects of care” constituting valued entities within the mindset – encompassing people, places, goals, or events – prompt cognitive processes that strive to optimize and prolong the existence of these cherished values. This optimization entails utilizing information stored in the mind and information acquired from the surrounding environment [40,7]. It’s essential to note that while climate change knowledge resides in the mind, it may not necessarily be a part of the mindset. Nevertheless, it can serve as a resource for cognitive processes aimed at preserving the existence of these “objects of care”.

When stakeholders are not at all concerned about climate change, they might perceive no “objects of care” threatened by climate change, or they do not believe in climate change (i.e., climate change denialists) (McCright and Dunlap, 2011; Vardy et al., 2017). In either case, they have different mindsets compared to stakeholders concerned about climate change. Such mindsets might subsequently influence their information processes to prioritize their other interests, such as livelihoods, but not information relevant to climate change reduction. For those people, climate change knowledge might be optimized to reject information about climate change reduction efforts. This reasoning might explain why, among stakeholders perceiving the benefits of marine and coastal ecosystems in climate change reduction, people with no concern about climate change have significantly lower support for marine protection when their knowledge increases. From the perspective of these people, protecting marine seems to be not valuable as they perceive no “objects of care” threatened by climate change or do not believe in climate change. Hence, they might utilize their climate change knowledge to avoid supporting marine protection, which can even incur costs to them. For stakeholders with no concern and knowledge but still perceiving the benefits of marine and coastal ecosystems in reducing climate change, they might have limited alternatives other than supporting marine protection, as it seems to be the most beneficial option.”

Similar to the first example, we included a quotation from “Light and Free” at the beginning of the paper to foreground its intellectual inspiration and to function as a cognitive catalyst for readers. Further details on how the Absurdist Approach was applied can be found in the study published in *Journal of Environmental Studies and Sciences* [29]

## Final remarks and limitations

In general, the combination of GITT and *Wild Wise Weird*’s hypothetical space of absurdist arguments forms the epistemic foundation for what we call the Absurdist Approach to Unveiling Paradoxical Thinking. This approach carries significant epistemological and methodological value in scientific reasoning, particularly in assessing the plausibility of research findings within today’s era of information and data abundance. By integrating *Wild Wise Weird*’s artistic and reflective paradoxes with GITT’s formal reasoning structure, the method establishes a bridge between analytical modeling and interpretive reflection—between the rigor of data analysis and humanistic values.

When formulating models or interpreting research findings—whether quantitative or qualitative—this approach allows scholars to perceive both the strengths and limitations of their analyses [[61], [64]]. This is because it integrates the principles of informational interactions from GITT with the examination of logical paradoxes that may emerge through the counter-assumptive propositions found in *Wild Wise Weird*. In other words, the method not only enhances the interpretive capacity of models and results, especially in cases involving seemingly contradictory or low-probability findings, but also fosters intellectual humility and epistemic moderation among scholars. Through this approach, scientific inquiry becomes less concerned with arriving at definitive conclusions and more oriented toward cultivating awareness of uncertainty, complexity, and relational interdependence. Such epistemological humility helps to reduce overgeneralization while enabling the appreciation of subtle, rare, or emergent patterns that may carry profound insights across diverse fields, including but not limited to psychology, sociology, healthcare, environmental studies, politics, and economics.

Nevertheless, this method also carries several limitations. First, the integration of literary and philosophical paradoxes remains inherently interpretive and thus context-dependent; its insights rely on the reader’s cultural, cognitive, and emotional receptivity. This subjectivity may challenge the standardization expected in empirical sciences. Second, while the absurdist approach enriches reasoning depth, it may introduce conceptual ambiguity if not carefully operationalized—particularly when translating allegorical insights into analytical frameworks. Thus, such imaginative allegorical insights should be carefully expressed through the dynamic and flexible explanation of GITT. Third, as an epistemic bridge between science and art, it demands interdisciplinary literacy that many researchers may not yet possess, potentially limiting its accessibility and application.

Despite these limitations, the absurdist approach represents a promising epistemological innovation. It invites scholars to look beyond data (in the usual sense), to perceive the nuanced interplay between reason and absurdity, and to appreciate how low-probability events and phenomena—often hidden beneath apparent contradictions—can illuminate the deeper structure of human understanding.

## Ethics statements

Not applicable.

## CRediT authorship contribution statement

**Minh-Hoang Nguyen:** Conceptualization, Formal analysis, Investigation, Resources, Writing – original draft, Writing – review & editing, Supervision, Project administration. **Manh-Tung Ho:** Resources, Writing – original draft.

## Declaration of competing interest

The authors declare that they have no known competing financial interests or personal relationships that could have appeared to influence the work reported in this paper.

## Data Availability

No data was used for the research described in the article.
